# In Vitro Protein Disappearance of Raw Chicken as Dog Foods Decreased by Thermal Processing, but Was Unaffected by Non-Thermal Processing

**DOI:** 10.3390/ani11051256

**Published:** 2021-04-27

**Authors:** Hansol Kim, Ah Hyun Jung, Sung Hee Park, Yohan Yoon, Beob Gyun Kim

**Affiliations:** 1 Department of Animal Science and Technology, Konkuk University, Seoul 05029, Korea; sunlight1995@naver.com; 2 Department of Food Science and Technology, Seoul National University of Science and Technology, Seoul 01811, Korea; ah.jung@seoultech.ac.kr (A.H.J.); sunghpark@seoultech.ac.kr (S.H.P.); 3 Department of Food and Nutrition, Sookmyung Women’s University, Seoul 04310, Korea; yyoon@sookmyung.ac.kr; 4 Risk Analysis Research Center, Sookmyung Women’s University, Seoul 04310, Korea; 5 Monogastric Animal Feed Research Institute, Konkuk University, Seoul 05029, Korea

**Keywords:** chicken meat, dogs, in vitro ileal disappearance, non-thermal processing, pasteurization, thermal processing

## Abstract

**Simple Summary:**

Chicken meat is widely used as a dog food due to its high nutritional values and palatability. Pasteurization is important to ensure the safety of chicken meat: thermal processing and non-thermal processing including high-pressure processing, ultraviolet-light emitting diode radiation, electron-beam irradiation, and gamma-ray irradiation. The influence of these pasteurization methods on nutrient digestibility is of interest. In the present work, the effects of thermal and non-thermal processing methods on protein digestibility of chicken meat were measured using in vitro assays. Protein digestibility of chicken meat was decreased by high-temperature processing at 70, 90, and 121 °C. However, non-thermal processing methods including high-pressure processing, ultraviolet-light emitting diode radiation, electron-beam irradiation, and gamma-ray irradiation did not affect protein digestibility of chicken meat. The present study indicates that nutritional values of chicken meat were maintained when non-thermal processing methods are used whereas they were decreased by thermal processing methods.

**Abstract:**

The objectives of the present study were to determine the influence of thermal and non-thermal processing procedures on in vitro ileal disappearance (IVID) of dry matter (DM) and crude protein (CP) in chicken meat as dog foods using 2-step in vitro assays. In thermal processing experiments, IVID of DM and CP in chicken meat thermally processed at 70, 90, and 121 °C, respectively, with increasing processing time was determined. For non-thermal processing experiments, IVID of DM and CP in chicken meat processed by high-pressure, ultraviolet-light emitting diode (UV-LED), electron-beam, and gamma-ray was determined. Thermal processing of chicken meat at 70, 90, and 121 °C resulted in decreased IVID of CP (*p* < 0.05) as heating time increased. In non-thermal processing experiment, IVID of CP in chicken meat was not affected by high-pressure processing or UV-LED radiation. In vitro ileal disappearance of CP in electron-beam- or gamma-ray-irradiated chicken meat was not affected by the irradiation intensity. Taken together, ileal protein digestibility of chicken meat for dogs is decreased by thermal processing, but is minimally affected by non-thermal processing methods.

## 1. Introduction

Animal-originated protein ingredients are widely used in dog foods as a source of amino acids (AA) to meet the requirements for maintenance and growth and as a source of flavors [1]. Raw chicken meat is one of the most representative animal-originated protein ingredients that can be used in extruded dog foods [2]. Chicken meat is potentially contaminated with pathogenic bacteria including *Salmonella*, *Campylobacter*, *Staphylococcus aureus*, *Escherichia coli*, and *Listeria* during slaughter, packaging, transportation, and storage [3,4,5]. Thus, the necessity of pasteurization increases to ensure food safety and hygiene of animal-originated protein ingredients. Thermal processing has been widely used for pasteurization of animal-originated protein ingredients [6], which causes problems in the organoleptic property and protein digestibility due to Maillard reaction, denaturation, and aggregation of proteins in the animal-originated protein ingredients [7,8]. To prevent these detrimental effects of thermal processing on animal-originated protein ingredients, non-thermal pasteurization methods are available including high-pressure processing, ultraviolet-light emitting diode (UV-LED) radiation, electron-beam irradiation, and gamma-ray irradiation [9,10]. The detrimental effects of thermal processing on protein digestibility of animal-originated protein ingredients have been reported [7,11]. However, information on the influence of non-thermal pasteurization on protein digestibility in chicken meat fed to dogs is very limited.

In an in vitro experiment conducted by Bax et al. [7], the influence of thermal processing of pork loin muscle at 70, 100, and 140 °C for 30 min on in vitro nutrient digestibility was investigated, and the authors found that the nutrient digestibility was reduced by heat treatment. Similarly, Sante-Lhoutellier et al. [12] also reported that the proteolysis rate of bovine muscle proteins by pepsin decreased as the cooking time increased at 100 °C. In an in vivo digestibility experiment employing cecectomized roosters, Wang et al. [11] reported that thermal processing of meat and bone meal at temperatures ranging from 96 to 152 °C for 15 to 240 min resulted in decreased digestibility of AA. 

Nutrients are digested and absorbed mostly in the stomach and small intestine of dogs [13]. Ileal digestibility values for AA are more accurate than total tract digestibility values due to the potential influence of hindgut fermentation on crude protein (CP) and AA [14]. However, the determination of ileal digestibility of CP and AA in dogs requires a surgery to insert a cannula at the end of the distal ileum. The ileal cannulation procedures are practically not applicable to dogs due to the problems including high costs and animal welfare issues [13]. Alternatively, in vitro procedures are inexpensive, time-saving, and non-invasive methods compared with in vivo experiments. In vitro assays are widely used to determine nutrient utilization in ingredients [7,15] and extruded diets [16,17] for dogs and feed ingredients [18,19,20,21,22,23,24] and diets [25,26] for pigs based on the high correlation between in vivo nutrient digestibility and in vitro nutrient disappearance [16,17,25,26,27]. 

The lack of data on the effects of non-thermal pasteurization on protein digestibility of meat as a dog food is a research gap. To bridge this research gap, therefore, the objectives of the present study were to determine the influence of thermal and non-thermal processing methods on ileal digestibility of dry matter (DM) and CP of chicken meat using in vitro procedures. We hypothesized that in vitro ileal disappearance (IVID) of CP in chicken meat was affected by thermal processing, but not by non-thermal processing.

## 2. Materials and Methods

### 2.1. Pasteurization Procedures of Chicken Meat 

Frozen chicken meat was thawed for 24 h at 4 °C before thermal and non-thermal processing. All processing procedures were conducted in triplicate. In thermal processing experiments, chicken meat was divided into 45 equal portions followed by individual packaging in a nylon-polyethylene bag using a vacuum packaging machine (Airfree, Postech Co. Ltd., Gyeongsangnam-do, Korea). Thirty samples were then processed at 70 °C in Experiment (Exp.) 1 and 90 °C in Exp. 2 for five processing durations (0, 1, 15, 30, and 60 min) using a continuously thermo-controlled water bath (HB-205 sw, Hanbaek Scientific Co., Gyeonggi-do, Korea). In Exp. 3, fifteen remaining samples were pasteurized by autoclaving at 121 °C and 1.2 atm for 0, 1, 4, 7, and 15 min.

In non-thermal processing experiments, high-pressure processing of chicken meat was performed (Exp. 4) on approximately 50 g of meat in a polyethylene vacuum packaging bag (150 mm × 150 mm) using a vacuum packer (FM5460-071, Food Saver, CA, USA). To prevent the rupture of the polyethylene vacuum packaging bag due to high pressure, the secondary packaging was carried out with a polyethylene vacuum packaging bag (200 mm × 150 mm). A high-pressure treatment was exposed to the samples using a 300-litter industrial scale high-pressure treatment equipment (HP 300, Hyperbaric, Burgos, Spain). The processing conditions were 1, 3, 5, and 7 min under a constant pressure of 500 MPa. The high-pressure processed samples were refrigerated to 4 °C to prevent temperature rise due to adiabatic heating during high-pressure processing. After high-pressure processing, samples were rapidly cooled down in the ice and water mixture.

For UV-LED radiations (Exp. 5), chicken meat samples (approximately 50 g each) were placed and sealed in a transparent polyethylene bag (177 mm × 188 mm). The 405-nm UV-LED A lamp (12 Watt, SWL-V2650, Sunwave, Suwon, Korea) emitted light to the samples. Five UV-LED A lamps were placed with 100-mm distance among lamps in a 4 °C refrigeration system (600 mm × 600 mm). The lamps were located 50 mm above the samples. In this way, all meat samples were uniformly exposed to the UV-LED light. A proportional-integral-derivative controller (ITC-100 VH, Shenzhen Inkbird Tech. Co., Shenzhen, China) was used to prevent a temperature over-rise during UV-LED emission on the chicken meat in the refrigeration system. The intensity dose of UV-LED emission was 0.00156 W/cm^2^ which was measured through spectrometer (StellarNet BLK-C, Stellar Net Inc., FL, USA). The samples were exposed to UV-LED A light for 30, 60, 90, and 120 min which corresponds to dose of 2.8, 5.6, 8.4, and 11.2 J/cm^2^.

Electron-beam (Exp. 6) and gamma-ray (Exp. 7) irradiation procedures were conducted using facilities of Greenpia Technology (Yeoju-si, Gyeonggi-do, Korea). A 50 g of chicken meat was packaged into transparent polyethylene pouch (177 mm × 188 mm) for electron-beam and gamma-ray irradiation. Electron-beam irradiation was conducted using a high-intensity electron accelerator (Rhodotron, TT-200, Ion Beam Applications s.a., Louvain-la-Neuve, Belgium) at 3, 5, 7, and 10 kGy. In the gamma-ray treatments, chicken meat samples were irradiated at 3, 5, 7, and 10 kGy using a cobalt gamma irradiation system (Gamma FIT, Nordion Inc., Ottawa, Canada).

### 2.2. Two-Step In Vitro Procedures and Chemical Analyses

All thermal and non-thermal processed meat samples from the 7 experiments were stored at 4 °C until chemical analyses and in vitro assays. A 2-step in vitro assay was conducted to determine IVID of DM and CP [16] in 3 samples per treatment following pasteurization procedures. The in vitro procedures were performed independently for each experiment. Briefly, 2 g of each chicken meat sample that was exposed to thermal or non-thermal processing was transferred into 100-mL conical flasks. A 25 mL of sodium phosphate buffer solution (0.1 M; pH 6.0) and 10 mL of HCl (0.2 M; pH 0.7) were added to each flask. To simulate digestion conditions in the stomach of a dog, the pH was adjusted 2.0, and 1 mL of freshly prepared pepsin solution (10 mg/mL; ≥250 units/mg solid, P7000, pepsin from porcine gastric mucosa, Sigma-Aldrich, St. Louis, MO, USA) was added to the samples. Test flasks were incubated in a shaking incubator at 39 °C for 2 h. To simulate digestion conditions in the small intestine, 10 mL of sodium phosphate buffer solution (0.2 M; pH 6.8) and 5 mL of NaOH (0.6 M; pH 13.8) were added to each flask and the pH was adjusted to 6.8. Thereafter, 1 mL of freshly prepared pancreatin solution (100 mg/mL; 4 × USP, P1750, pancreatin from porcine pancreas, Sigma-Aldrich, St. Louis, MO, USA) was added. After incubation in a shaking incubator at 39 °C for 4 h, 5 mL of 20% sulfosalicylic acid solution was added and samples were left at room temperature for 30 min to precipitate the indigestible protein. The samples were then filtered through pre-dried and pre-weighed glass filter crucibles (Filter Crucibles CFE Por. 2, Robu, Hattert, Germany) containing 500 mg of Celite. Test flasks were rinsed twice with 1% sulfosalicylic acid solution, and 10 mL of 95% ethanol and 10 mL of 99.5% acetone were added twice to the glass filter crucibles. Glass filter crucibles with undigested residues and Celite were dried at 80 °C for 24 h. After conducting the 2-step procedure, undigested residues on filter crucibles were collected for analyzing CP contents to calculate IVID of CP. During each 2-step procedure, a blank was also included. The residues on the filter crucible in the blank were collected and analyzed for DM and CP concentrations to correct the DM and CP contents in the residues that were not originated from meat samples.

Dry matter (method 950.46) [28] concentrations in chicken meat were determined. Crude protein (method 976.05) [28] concentrations in chicken meat and residue were determined.

### 2.3. Calculations and Statistical Analyses

The IVID of DM was calculated using the following equation:

IVID of DM (%) = [DM_Meat_ − (DM_UR_ − DM_Blank_)] ÷ DM_Meat_ (1)

where, DM_Meat_ (g) is the amount of thermal or non-thermal processed chicken meat as DM basis, DM_UR_ (g) is the amount of undigested residue after in vitro digestion procedures, and DM_Blank_ (g) is the amount of DM residue after in vitro digestion procedures in the blank. 

After the 2-step in vitro assay, the undigested residues and Celite were collected, weighed, and analyzed for CP. Then, IVID of CP was calculated using the following equation modified from Ha et al. [18]:

IVID of CP (%) = [(DM_Meat_ × CP_Meat_) − {(DM_UR_ × CP_UR_) − (DM_Blank_ × CP_Blank_)}] ÷ (DM_Meat_ × CP_Meat_) × 100 (2)

where, CP_Meat_, CP_UR_, and CP_Blank_ are the CP concentrations (%) expressed as DM basis in the thermal or non-thermal processed chicken meat, undigested residue, and blank, respectively.

Data for each experiment were analyzed using the MIXED procedure of the SAS (SAS Inst. Inc., Cary, NC, USA). The model included thermal or non-thermal processing as a fixed variable. The least squares mean was calculated for each treatment. Preplanned orthogonal polynomial contrasts were employed to analyze linear and quadratic effects of thermal, high-pressure, and UV-LED processing time and electron-beam and gamma-ray irradiation intensity on IVID of nutrients in chicken meat. Appropriate coefficients for unequally spaced processing times and irradiation intensities for each experiment were obtained using the interactive matrix language procedure of SAS. The statistical model of the current study is as follows:

Y_ij_ = μ + Τ_i_ + ε_ij_ (3)

where, Y_ij_ is the response variable, μ is the overall mean, Τ_i_ is the effect of thermal or non-thermal processing, and ε_ij_ is the error. The experimental unit was the mean of the duplicate analyses for each processed chicken meat sample, and an alpha level of 0.05 was used to determine statistical significance.

## 3. Results

### 3.1. Thermal Processing of Chicken Meat

In vitro ileal disappearance of DM in chicken meat thermally processed at 70 °C was not affected by heating time in Exp. 1 (Table 1). However, IVID of CP was decreased (*p* < 0.05; linear and quadratic) as heating time extended. In Exp. 2 and 3, IVID of DM and CP in chicken meat thermally processed at 90 °C and 121 °C decreased (*p* < 0.05; linear and quadratic) as heating time increased (Table 2 and Table 3).

### 3.2. Non-Thermal Processing of Chicken Meat

In Exp. 4, IVID of DM in high-pressure processed chicken meat decreased linearly (*p* < 0.001) as processing time increased (Table 4). However, IVID of CP in high-pressure processed chicken meat was not affected by processing time. In Exp. 5, IVID of DM and CP in UV-LED-radiated chicken meat was not affected by processing time (Table 5). In Exp. 6 and 7, IVID of DM and CP in electron-beam- and gamma-ray-irradiated chicken meat was not affected by the irradiation intensity (Table 6 and Table 7).

## 4. Discussion

Chicken meat, widely used in dog foods, is often contaminated with potentially pathogenic microorganisms [3,4,5]. Therefore, pasteurization of chicken meat for dog foods has gained considerable interest [29]. While it is well-known that thermal processing effectively destructs pathogens and parasites [5,6], heating negatively affects the secondary and tertiary structures of proteins in raw meat [5,30]. To prevent heat-induced denaturation and aggregation of proteins in chicken meat, non-thermal pasteurization methods are available including high-pressure processing [9,31,32,33], UV-LED radiation [9,33,34,35], electron-beam irradiation [10,36,37], and gamma-ray irradiation [9,10,33,37]. However, high-pressure processing can stimulate physicochemical changes in texture, color, sensorial alterations, and pH in chicken breast fillet [38]. In addition, UV-LED radiation potentially has detrimental effects on nutritional values in meat [39]. Irradiation of electron-beam and gamma-ray on meat also can cause AA-protein, protein-protein, or lipid-protein aggregates [40]. To our knowledge, however, information on the effects of non-thermal processing on nutrient digestibility of chicken meat for dogs is very limited. In the present work, to bridge this gap, the effects of thermal and non-thermal processing on chicken meat were measured using an in vitro assay.

In the present work, DM and CP concentrations in chicken meat without thermal or non-thermal processing (control) ranged from 28.7 to 32.2% and 17.5 to 18.9% (as-is basis), respectively. These values are comparable to the previously reported values in the literature [41]. Akramzadeh et al. [42] reported that similar CP concentration (17.7%) in mechanically deboned whole chicken meat, but the DM concentration in the present work was greater than the DM concentration in mechanically deboned whole chicken meat (26.7%). In the other experiment [43], the DM and CP concentrations in thigh meat (25.2% for DM and 19.0% for CP, as-is basis) and breast meat (28.4% for DM and 21.8% for CP, as-is basis) deviated from the values in the present work. The factors that affect the nutrient composition of chicken meat include broiler nutrition, management, biochemical changes, carcass temperature, pre-slaughter factors, and genetics [44]. In addition, chicken thigh meat and breast meat are mostly consisted of muscles that are consisted of 75, 20, and 3% of moisture, protein, and fat, respectively [30], whereas whole ground chicken meat used in the present work included bone and skin as well as muscular meat. This mainly explains inconsistent DM and CP concentrations in broiler meat among studies including the present work. In all experiments, the IVID of DM and CP in the control groups were very constant with minor deviation in Exp. 5. However, the relatively low IVID of DM and CP in the control group of Exp. 5 compared with other experiments does not prohibit assessing the influence of thermal and non-thermal processing methods on the digestibility in each experiment.

In the present work, the decreased IVID of DM and CP by thermal treatment, particular at high temperatures, is likely due to heat-induced protein oxidation, denaturation, aggregation, and hydrophobicity in meat protein. Bax et al. [7] also reported that heat treatment caused protein oxidation, protein aggregation, and reduced protein digestibility by pepsin. Denaturation is conformational changes of secondary and tertiary protein structures [30]. Aggregation is a process of protein monomers interacting to form fibrils and amorphous aggregates [45]. Denaturation of proteins occurs during the pasteurizing process at over 70 °C [7]. At higher temperatures, proteins are further modified by oxidation [7], which promotes heat-induced protein aggregation, increases protein hydrophobicity, and alters secondary and tertiary structure [5,30,46]. In the study of Sante-Lhoutellier et al. [47], protein hydrophobicity was negatively correlated with exogenous protein-digesting enzyme activities. Therefore, the decreased IVID of CP by thermal processing in the present work is likely due to heat-induced physicochemical changes in meat protein. The reduced CP digestibility by thermal processing in the present work is in agreement with other non-ruminant experiments. In cecectomized roosters assays by Wang et al. [11], the digestibility of Lys, Cys, Met, and Thr in meat and bone meal processed under high temperature was less than that processed under low temperature. Although not an animal-based protein ingredient, the ileal digestibility of CP and AA in soybean meal also decreased as the autoclaving time increased to 30 min at 125 °C in pigs [48].

Pressures above 200 MPa during meat processing can cause protein aggregation as a consequence of the protein unfolding [32]. High-pressure processing has also been reported to cause denaturation and aggregation in myofibrillar proteins from bovine muscle [49]. In the present work, the decreased IVID of DM in high-pressure processed chicken meat was possibly attributed to the denaturation and aggregation of proteins in chicken meat due to the high-pressure processing condition of 500 MPa. In the study of Hu et al. [50], in vitro AA digestibility of alpha-casein was decreased by the high-pressure processing with 600 MPa which was similar to the present work. However, specific reasons for the negative effects of high-pressure processing on IVID of DM but not on IVID of CP are unknown.

Data on the effects of UV-LED radiation on the nutrient digestibility of meat are scarce. In vitro ileal digestibility of DM and CP in UV-LED-radiated chicken meat was not affected as the radiation time increased in the present work, whereas in vitro AA digestibility was decreased in alpha-casein by UV-LED radiation in a study by Hu et al. [50]. This discrepancy may be attributed to different animal-origin food ingredients, in vitro methods, and UV-LED radiation conditions. Hu et al. [50] used alpha-casein that was isolated from fresh bovine milk using the urea differential precipitation method and used different UV-LED radiation dose and time from the present work.

In the present work, electron-beam or gamma-ray irradiation up to 10 kGy did not affect IVID of DM and CP of chicken meat. Similarly, Park et al. [37] reported that hardness, color, chewiness, and taste of beef sausage patties were not affected by the electron-beam or gamma-ray irradiation up to 0, 5, and 10 kGy. Farkas [10] also suggested that irradiation minimally affects chemical compositions in foods. Although not an animal-originated food ingredient, electron-beam irradiation on lotus seed at up to 15 kGy did not affect in vitro CP digestibility, but electron-beam irradiation at over 15 kGy resulted in reduced CP digestibility [51]. Thus, the intensity of irradiation used in the present work appears to be rather low to induce chemical changes or nutrient digestibility reductions in chicken meat.

The temperature and duration of thermal processing and the intensity and duration of non-thermal processing employed in the present work were similar to the methods that are generally used for food pasteurization [5,9,10,33,37]. For this reason, the treatment times and intensities were inconsistent among the experiments. Additionally, each experiment was performed independently. Thus, we did not pool the data from seven experiments for addressing the interaction between the processing method and treatment time or intensity. In the statistical analysis procedures, as each experiment was regarded as independent, the result descriptions were limited to comparisons within each experiment.

## 5. Conclusions

Based on the present 2-step in vitro assays mimicking the stomach and small intestine digestion and absorption of dogs, in vitro ileal disappearance of protein in chicken meat was decreased by thermal processing at 70 and 90 °C for up to 60 min, and at 121 °C for up to 15 min. However, non-thermal processing methods including high-pressure processing under 500 MPa for up to 7 min, ultraviolet-light emitting diode radiation at 0.00156 W/cm^2^ for up to 120 min, electron-beam irradiation with an intensity of up to 10 kGy, and gamma-ray irradiation with an intensity of up to 10 kGy did not affect in vitro ileal disappearance of protein in chicken meat. Taken together, non-thermal processing methods do not cause detrimental effects on protein digestibility of chicken meat. As the present conclusion is limited to only chicken meat, further research is warranted to investigate the influence of non-thermal processing methods on the nutrient digestibility of other dog foods. In vivo tests possibly employing cecectomized roosters are also warranted to validate the effects of non-thermal processing on protein digestibility in dog foods.

## Figures and Tables

**Table 1:** Dry matter and crude protein concentrations in chicken meat thermally processed at 70 °C (as-is basis) using water bath and the effects of heating time on in vitro ileal disappearance of dry matter and crude protein of chicken meat thermally processed at 70 °C (Exp. 1) ^1^.

Item, %	Temperature, °C:	70					SEM	*p*-Value	
	Time, min:	0	1	15	30	60		Linear	Quadratic
Dry matter		29.8	28.9	30.3	31.1	31.5	-	-	-
Crude protein		18.3	19.0	18.6	19.1	22.0	-	-	-
In vitro ileal disappearance of dry matter		87.7	86.6	86.6	86.5	87.2	0.80	0.992	0.403
In vitro ileal disappearance of crude protein		86.7	86.5	84.8	85.0	85.3	0.41	0.025	0.014

SEM = standard error of the mean. ^1^ Each least squares mean represents 3 observations in duplicate.

**Table 2:** Dry matter and crude protein concentrations in chicken meat thermally processed at 90 °C (as-is basis) using water bath and the effects of heating time on in vitro ileal disappearance of dry matter and crude protein of chicken meat thermally processed at 90 °C (Exp. 2) ^1^.

Item, %	Temperature, °C:	90					SEM	*p*-Value	
	Time, min:	0	1	15	30	60		Linear	Quadratic
Dry matter		29.7	29.6	30.4	30.8	29.4	-	-	-
Crude protein		18.6	19.6	20.3	20.6	20.9	-	-	-
In vitro ileal disappearance of dry matter		87.3	84.5	83.3	78.3	77.5	0.60	<0.001	0.002
In vitro ileal disappearance of crude protein		85.2	83.5	82.6	77.4	74.7	0.44	<0.001	0.022

SEM = standard error of the mean. ^1^ Each least squares mean represents 3 observations in duplicate.

**Table 3:** Dry matter and crude protein concentrations in chicken meat autoclaved at 121 °C (as-is basis) and the effects of heating time on in vitro ileal disappearance of dry matter and crude protein of chicken meat autoclaved at 121 °C (Exp. 3) ^1^.

Item, %	Temperature, °C:	121					SEM	*p*-Value	
	Time, min:	0	1	4	7	15		Linear	Quadratic
Dry matter		30.3	36.6	34.9	37.2	38.6	-	-	-
Crude protein		17.7	23.0	25.0	25.2	26.0	-	-	-
In vitro ileal disappearance of dry matter		86.2	78.6	77.9	74.9	73.8	0.86	<0.001	<0.001
In vitro ileal disappearance of crude protein		84.6	70.4	69.8	66.1	64.6	0.74	<0.001	<0.001

SEM = standard error of the mean. ^1^ Each least squares mean represents 3 observations in duplicate.

**Table 4:** Dry matter and crude protein concentrations in high-pressure processed chicken meat (as-is basis) and effects of processing time on in vitro ileal disappearance of dry matter and crude protein of high-pressure processed chicken meat (Exp. 4) ^1,2^.

Item, %	Processing Time, min					SEM	*p*-Value	
	0	1	3	5	7		Linear	Quadratic
Dry matter	32.2	31.9	31.6	33.2	32.2	-	-	-
Crude protein	18.9	20.3	20.5	19.5	18.9	-	-	-
In vitro ileal disappearance of dry matter	88.8	87.2	87.5	87.2	85.0	0.45	<0.001	0.253
In vitro ileal disappearance of crude protein	85.9	85.5	86.7	84.4	84.1	0.85	0.105	0.339

SEM = standard error of the mean. ^1^ Each least squares mean represents 3 observations in duplicate. ^2^ The high-pressure processing was conducted under a constant pressure of 500 MPa.

**Table 5:** Dry matter and crude protein concentrations in ultraviolet-light emitting diode (UV-LED)-radiated chicken meat (as-is basis) and effects of radiation time on in vitro ileal disappearance of dry matter and crude protein of UV-LED radiated chicken meat (Exp. 5) ^1,2^.

Item, %	Radiation Time, min					SEM	*p*-Value	
	0	30	60	90	120		Linear	Quadratic
Dry matter	31.8	32.3	30.7	31.5	32.4	-	-	-
Crude protein	18.3	18.2	18.3	17.9	18.0	-	-	-
In vitro ileal disappearance of dry matter	80.5	80.9	80.6	82.0	80.1	1.54	0.962	0.613
In vitro ileal disappearance of crude protein	79.9	80.0	81.9	81.9	78.8	1.00	0.935	0.050

SEM = standard error of the mean. ^1^ Each least squares mean represents 3 observations in duplicate. ^2^ The intensity dose of UV-LED emission was 0.00156 W/cm^2^.

**Table 6:** Dry matter and crude protein concentrations in electron-beam-irradiated chicken meat (as-is basis) and effects of irradiation intensity on in vitro ileal disappearance of dry matter and crude protein of electron-beam-irradiated chicken meat (Exp. 6) ^1^.

Item, %	Irradiation Intensity, kGy					SEM	*p*-Value	
	0	3	5	7	10		Linear	Quadratic
Dry matter	30.0	28.7	29.8	30.3	28.6	-	-	-
Crude protein	17.5	17.8	18.3	18.5	18.9	-	-	-
In vitro ileal disappearance of dry matter	87.7	88.0	88.9	85.2	87.1	0.91	0.234	0.831
In vitro ileal disappearance of crude protein	85.6	86.1	85.8	85.0	85.9	0.55	0.948	0.857

SEM = standard error of the mean. ^1^ Each least squares mean represents 3 observations in duplicate.

**Table 7:** Dry matter and crude protein concentrations in gamma-ray-irradiated chicken meat (as-is basis) and effects of irradiation condition on in vitro ileal disappearance of dry matter and crude protein of gamma-ray-irradiated chicken meat (Exp. 7) ^1^.

Item, %	Irradiation Intensity, kGy					SEM	*p*-Value	
	0	3	5	7	10		Linear	Quadratic
Dry matter	31.5	31.0	31.1	31.2	30.6	-	-	-
Crude protein	18.5	17.5	18.3	18.4	19.1	-	-	-
In vitro ileal disappearance of dry matter	86.7	86.0	87.1	86.6	84.8	1.00	0.282	0.347
In vitro ileal disappearance of crude protein	85.2	86.5	85.4	85.8	85.5	0.74	0.990	0.494

SEM = standard error of the mean. ^1^ Each least squares mean represents 3 observations in duplicate.

## Data Availability

The data presented in this study are available on request from the corresponding author.
